# Clinicopathological and prognostic significance of mTOR and phosphorylated mTOR expression in patients with esophageal squamous cell carcinoma: a systematic review and meta-analysis

**DOI:** 10.1186/s12885-016-2940-7

**Published:** 2016-11-11

**Authors:** Shuangjiang Li, Zhiqiang Wang, Jian Huang, Shan Cheng, Heng Du, Guowei Che, Yong Peng

**Affiliations:** 1 Department of Thoracic Surgery, West China Hospital, Sichuan University, Guoxue Alley No. 37, Chengdu, China; 2 Department of Sonography, West China Hospital, Sichuan University, Guoxue Alley No. 37, Chengdu, China; 3 State Key Laboratory of Biotherapy and Cancer Center, West China Hospital, Sichuan University, Guoxue Alley No. 37, Chengdu, China

**Keywords:** Mammalian target of rapamycin (mTOR), Esophageal squamous cell carcinoma, Prognosis, Systematic review, Meta-analysis

## Abstract

**Background:**

Mammalian target of rapamycin (mTOR) is a serine/threonine protein kinase responsible for regulating ribosomal biogenesis and protein synthesis. Dysregulation of mTOR contributes to tumorigenesis, angiogenesis, cellular growth and metastasis but its roles in esophageal squamous cell carcinoma (ESCC) are controversial. Therefore, the objective of this study is to evaluate the prognostic and clinicopathological significance of mTOR/p-mTOR expression in ESCC.

**Methods:**

Literature retrieval was conducted by searching PubMed, EMBASE and the Web of Science for full-text papers that met our eligibility criteria. Odds ratio (OR) and hazard ratio (HR) with 95 % confidence interval (CI) served as the appropriate summarized statistics for assessments of clinicopathological and prognostic significance, respectively. Cochrane Q-test and I^2^-statistic were adopted to estimate the heterogeneity level between studies. Potential publication bias was detected by Begg’s test and Egger’s test.

**Results:**

A total of 915 ESCC patients from nine original articles were included into this meta-analysis. The pooled analyses suggested that mTOR/p-mTOR expression was significantly correlated with the unfavorable outcomes of differentiation degree (OR: 2.63; 95 % CI: 1.71–4.05; *P* = 0.001), tumor invasion (OR: 1.48; 95 % CI: 1.02–2.13; *P* = 0.037), TNM stage (OR: 2.25; 95 % CI: 1.05–4.82; *P* = 0.037) and lymph node metastasis (OR: 1.82; 95 % CI: 1.06–3.11; *P* = 0.029), but had no significant relationship to the genders (OR: 0.81; 95 % CI: 0.50–1.32; *P* = 0.396). Moreover, mTOR/p-mTOR expression could independently predict the worse overall survival (HR: 2.04; 95 % CI: 1.58–2.62; *P* < 0.001), disease-free survival (HR: 2.39; 95 % CI: 1.64–3.49; *P* < 0.001) and cancer-specific survival (HR: 1.62; 95 % CI: 1.18–2.23; *P* = 0.003) of patients with ESCC. Such prognostic value of mTOR was not substantially altered by further subgroup analyses.

**Conclusions:**

Positive expression of mTOR and p-mTOR was significantly associated with the unfavorable conditions on the depth of tumor invasion, TNM stage, differentiation degree and lymph node metastasis. mTOR and p-mTOR could serve as a valuable predictor for the poor prognosis of ESCC. More high-quality worldwide studies performing a multivariate analysis based on larger sample size are urgently required for further verifying and modifying our findings in the future.

**Electronic supplementary material:**

The online version of this article (doi:10.1186/s12885-016-2940-7) contains supplementary material, which is available to authorized users.

## Background

Esophageal squamous cell carcinoma (ESCC) is one of the highly aggressive cancers. It has become a worldwide challenge to human health, particularly to the peoples in developing countries [1]. According to the latest authoritative estimations in China, the incidence of ESCC ranks the fourth in all cancers, with the rate of 22.14 cases per 100,000 people. Moreover, the mortality of ESCC also ranks the fourth in all cancers, with a crude rate of 16.77 cases per 100,000 people [2, 3]. The five-year survival rate of operable ESCC ranges from 10 % to 36 %, suggesting its current poor prognosis [4, 5]. During the last decade, advanced surgical techniques, anesthetic techniques and perioperative managements have dramatically improved the feasibility and safety of esophagectomy but hardly benefited the prognosis of ESCC [4]. The possible main reason may be the detectable regional and distant metastasis in most of the patients with ESCC [6, 7]. The local-regional recurrence rate after esophagectomy ranges from 20.5 % to 43 %, which can also cause adverse effects on the survival outcomes of ESCC [8–10].

Given such concerns, identifying a group of novel biomarkers efficiently promising the prognostic and clinicopathological characteristics of ESCC is in urgent need. In recent years, the phosphatidylinositol 3-kinase/v-akt murine thymoma viral oncogene homolog 1/mammalian target of rapamycin pathway (PI3K/Akt/mTOR pathway) has emerged as one potential candidate on serving as a therapeutic target of cancers [11]. As a key component of this signaling pathway, mTOR is also known as “FK506 binding protein 12-rapamycin associated protein 1” and serves as a serine/threonine protein kinase responsible for regulating protein synthesis, ribosomal protein translation and cap-dependent translation [12]. In response to extracellular stimuli, mTOR is activated by the phosphorylation of Ser2448 through the PI3K/Akt/mTOR pathway, and it then activates the eukaryotic translation factor 4E (elF4E) and p70 ribosomal S6 kinase (p70S6 kinase) [12, 13]. mTOR consists of two independent functional complexes, mTORC1 and mTORC2, and the dysregulation of mTOR plays a crucial role in tumorigenesis, angiogenesis, cellular growth and metastasis [12, 14].

Nowadays, oncologists have increasingly focused on the potential of mTOR as an anticancer therapeutic target and evaluated its specific inhibitors in some phase I/II trials [15–17]. The potential prognostic value of mTOR and phosphorylated mTOR (p-mTOR) has also been extensively studied in a variety of cancers, including lung cancer [18], gastric cancer [19, 20], breast cancer [21], colorectal cancer [22, 23] and urological cancer [24]. Recently, many clinical reports have attempted to investigate the roles of mTOR and p-mTOR in ESCC but some controversial results are not well-interpreted. A consensus concerning the prognostic value of mTOR/p-mTOR expression and its relationship to some common clinicopathological characteristics of ESCC still remains a debate until now.

Limited sample availability in individual studies may result in negative bias risks on clarifying this pending issue accurately. Meta-analysis is generally regarded as a well-established method synthesizing the appropriate evidence from homogeneous studies to draw global conclusions [25–29]. Therefore, we carried out the current systematic review with meta-analysis to evaluate the prognostic and clinicopathological significance of mTOR/p-mTOR expression in patients with ESCC.

## Methods

### Protocol

No protocol had been previously published for this review. Our study was conducted in accordance with the Preferred Reporting Items for Systematic Reviews and Meta-Analyses (PRISMA) statement (PRISMA 2009 Checklist not shown) [30].

### Eligibility criteria

The following inclusion and exclusion criteria were established to determine the appropriate studies included into our meta-analysis.

#### Inclusion criteria

For the study designs, a quantitatively comparative analysis performed among the consecutive patients could be considered of eligibility.

For the participants, the target disease was ESCC, including all the clinical stages required for surgical procedures, neo-adjuvant and adjuvant therapies. No limitation was imposed for ages or genders.

For the interventions, the positive expression of mTOR/p-mTOR should be independently analyzed instead of collaborating with other biomarkers. Immunohistochemistry (IHC) was considered as the only eligible experimental method for mTOR/p-mTOR staining in ESCC specimens.

For the outcome measures, studies reporting any one of the following data in their results could be included into this meta-analysis. First, sufficient demographics or statistics should be available for the estimate of odds ratio (OR), relative risk (RR) and hazard ratio (HR) to determine the relationship between mTOR/p-mTOR expression and clinicopathological characteristics of ESCC. Second, any statistic evaluating the prognostic significance of mTOR/p-mTOR expression in ESCC was directly reported. Third, if no statistical result was conducted, the survival data with log-rank *P* value and Kaplan-Meier survival curves would also be considered of eligibility.

For the follow-ups, the key endpoints involved the overall survival (OS), disease-free survival (DFS) and cancer-specific survival (CSS). The follow-up period should be lasting for at least one year.

In addition, the most recent studies should be finally included if they were performed on overlapping patients. Only full-text papers published in peer-reviewed journals were finally included.

#### Exclusion criteria

Firstly, the following articles should be immediately excluded because of their irrelevant styles, including case reports, reviews, animal experiments, conference abstracts and letters. Secondly, a comparison of mTOR/p-mTOR expression between carcinomatous tissues and normal tissues was not considered. Thirdly, any continuous variable would not be included into quantitative synthesis. Fourth, positive expression of mTOR/p-mTOR was not stained by IHC.

### Search strategy

A comprehensive literature search for this meta-analysis was conducted between May 16, 2016 and May 21, 2016. No language or publication date restriction was imposed during the retrieval.

Two of our researchers were assigned to search three universal electronic databases, including PubMed, EMBASE (via Ovid interface) and the Web of Science (via campus network of Sichuan University), to identify the eligible studies published up to May 16, 2016. Consulting similar meta-analyses addressing on the prognostic value of biomarkers [25, 26, 28], we combined the following six key words with Boolean Operators (“AND” and “OR”), including four “esophageal cancer” terms and two “mTOR” terms, to formulate two search strings in each selected database. These key words are listed as follows:

(I) *Esophageal cancer terms:* “esophageal cancer”, “esophageal carcinoma”, “esophageal neoplasm” and “esophageal malignancy”;
(II) * mTOR terms:* “mammalian target of rapamycin” and “mTOR”.

Complete search details are outlined in the Additional file 1. Meanwhile, a manual search for the reference lists of retried studies was also conducted to identify any possibly included study with no duplication.

### Data collection

#### Process

We designed a Microsoft Office Excel spreadsheet to extract the basic information from included studies. The data collection process was developed by two of our researchers and cross-checked by another one reviewer.

#### Data items

The following details were collected from each included study:

(i) Publication data including authors, publication years, populations and languages;
(ii) Experimental data including study design, study period, investigating categories, experimental materials, detecting methods, IHC techniques (antibodies and dilution), positive-staining sites, cut-off values, endpoints and follow-up periods;
(iii) Demographic data including total sample size, genders, ages, the number of patients with positive and negative expression of mTOR/p-mTOR, the number of patients treated with neo-adjuvant induction therapy (NIT), and TNM stages of ESCC.
(iv) Statistical data including the outcome statistics with their extractions, and the corresponding statistical analysis methods (including univariate analysis and multivariate analysis).

### Risk of bias in individual studies

Newcastle-Ottawa Scale (NOS) was employed to quantify the quality levels of non-randomized studies [31]. Three perspectives including selection, comparability and exposure were considered for a semi-quantitative estimation. The “star system” with a maximum of 9 stars was used to grade all the included studies. We regarded 8–9 stars as good quality, 6–7 stars as fair quality, and lower than 6 stars as poor quality.

### Statistical analysis

All of the following steps of statistical analyses were accomplished by STATA 12.0 (STATA Corporation, College Station, TX, USA).

#### Summary measures

For the assessments of relationships between mTOR/p-mTOR expression and clinicopathological features of ESCC, OR with 95 % CI served as the appropriate summarized statistics. These OR outcomes were generally extrapolated from the reported demographic data [32]. If the relevant HR or RR was reported, we could immediately incorporate it into the meta-analysis.

For the assessments of prognostic value of mTOR/p-mTOR expression in ESCC, we determined the HR with 95 % CI to be the summarized estimates because HR was the only appropriate statistic compatible for both censoring and time-to-events [33]. It would be our first priority to incorporate the HR outcomes derived from multivariate analysis into quantitative synthesis because multivariate analysis using logistic regression or Cox proportional hazards model was generally used to eliminate the bias risks from other confounding factors in observational studies. If no multivariate statistic was available, we could extract the HR with 95 % CI from the reported survival data with log-rank *P* value according to a practical method described by Tierney et al. [34]. The formulas used for HR extractions are given as follows.

$$ \begin{array}{l}O-E=\frac{\sqrt{Total\  observed\  events\times Analyzed\  research\times Analyzed\  control}}{\left( Analyzed\  research+ Analyzed\  control\right)}\times \left(Z\  score\ for\ P\  value/2\right)\hfill \\ {}V=\frac{Total\  observed\  events\times Analyzed\  research\times Analyzed\  control}{{\left( Analyzed\  research+ Analyzed\  control\right)}^2}\hfill \\ {}HR=Exp\left(-\frac{O-E}{V}\right)\hfill \end{array} $$

Where O-E is the *log-rank Observed minus Expected events* and V is the *log-rank Variance* [34].

#### Synthesis of results

Both the Cochrane Q-test and I^2^-statistic were adopted for the estimate of heterogeneity level within this meta-analysis. Fine heterogeneity was defined by I^2^ < 50 % and *p* > 0.1, and a standard fixed-effect model test (Mantel–Haenszel method) would be required for quantitative synthesis. Otherwise, a random-effect model test (DerSimonian and Laird method) would be applied when a prominent heterogeneity was revealed by I^2^ ≥ 50 % or *p* ≤ 0.1 [35].

#### Additional analysis

Sensitivity analysis was conducted to examine the stability of all summarized outcomes, in which the impact of each study on the overall estimates could be detected by omitting the individual study sequentially. The robustness of our meta-analysis would be confirmed if there was no substantial variation between the adjusted pooled estimates and primary pooled estimates [36].

#### Publication bias

Both the Begg’s test and Egger’s test were collaborated to evaluate the potential publication bias between studies. On the one hand, the presence of bias was suggested by the visual symmetry of Begg’s funnel plot, in which log ORs or log HRs were plotted against their corresponding standard errors [37]. On the other hand, its significance was also suggested by Egger’s *p* value. Finally, a significant bias would be revealed by either visual asymmetry of Begg’s funnel plot or Egger’s *p* value < 0.05.

## Results

### Study selection

**Fig. 1:**
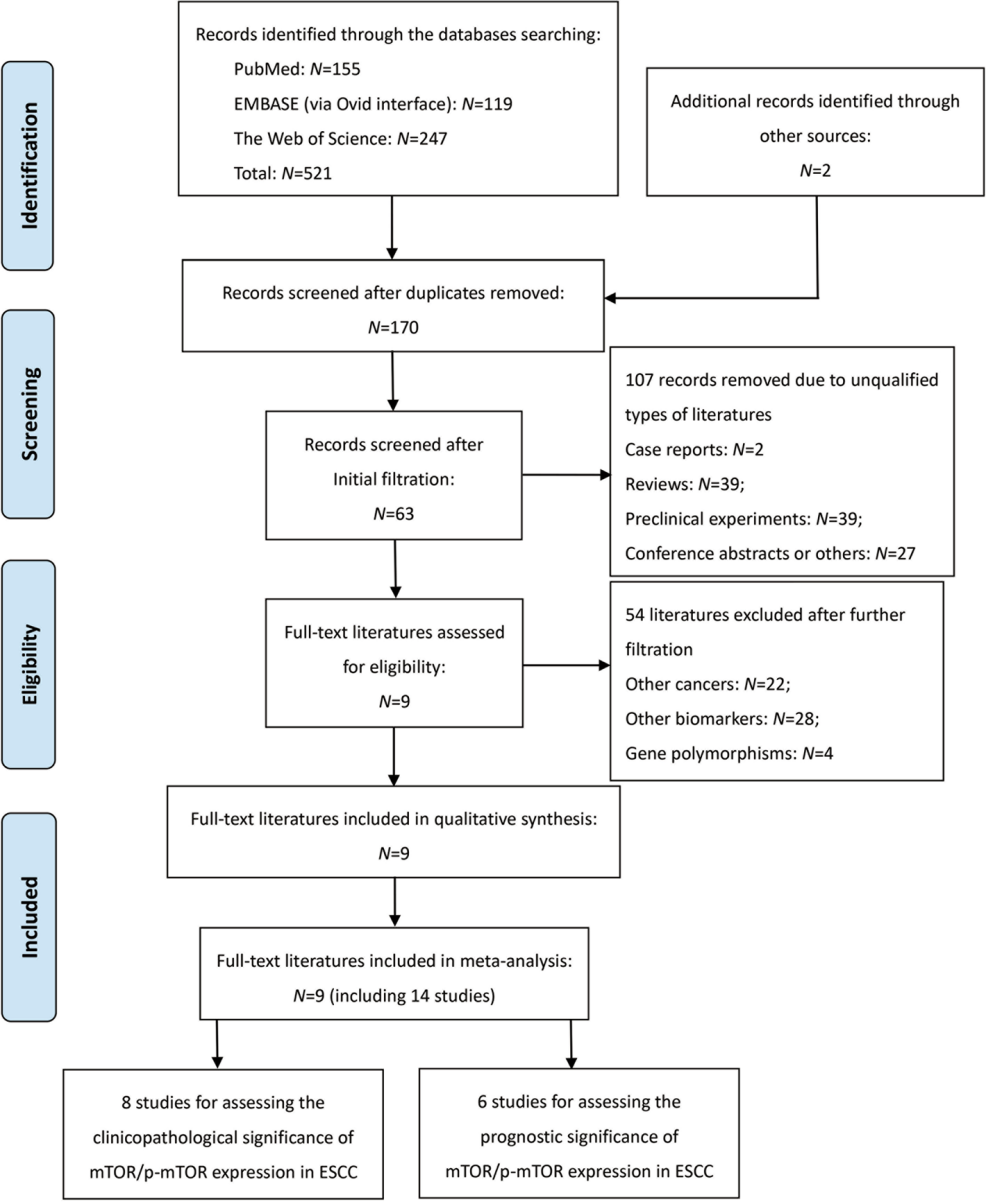
Preferred Reporting Items for Systematic Reviews and Meta-Analyses flow diagram for literature retrieval. ESCC, esophageal squamous cell carcinoma; mTOR, mammalian target of rapamycin; p-mTOR, phosphorylated mammalian target of rapamycin

The major procedures for literature retrieval was concisely summarized as a PRISMA diagram (Fig. 1). A total of 521 items of publications were primarily identified from the electronic databases, including 155 citations in PubMed, 119 citations in EMBASE and 247 citations in the Web of Science. In addition, a manual search of the reference lists also yielded two potentially relevant studies. After excluding 353 duplicates, the remaining 170 works entered into the initial filtration based on screening their titles and abstracts. Then, 107 of them were immediately excluded from the further filtration because of their irrelevant styles. By reading through the retrieved papers, 54 articles focusing on irrelevant issues were further excluded and the remaining nine articles were considered of possible eligibility. Finally, these nine studies were judged to meet all of the eligibility criteria and included into this meta-analysis [38–46].

### Study characteristics

Baseline characteristics for nine eligible articles are generalized in Tables 1 and 2.

**Table 1:** Baseline characteristics of included studies

Authors (Year)	Language	Populations	Study design	Study period	NOS	Categories		No. samples			Mean age (Years)	Genders (Male/Female)	NIT (Yes/No)	Stages
						CP features	Prognosis	Total	PE	NE				
Boone et al. (2008) [38]	English	Netherland	ROS	1989–2006	7	**✓**	**✗**	105	26	79	62.0	56/49	None	I-IV
Chen et al. (2010) [39]	Chinese	China mainland	ROS	2006–2007	7	**✓**	**✗**	62	33	29	NI	NI	None	NI
Chuang et al. (2015) [40]	English	Taiwan	ROS	NI	8	**✓**	**✓**	75	39	36	57.0	72/3	54/21	I-IV
Hirashima et al. (2010) [41]	English	Japan	ROS	1996–2006	9	**✓**	**✓**	143	71	72	63.8	126/17	None	I-III
Hou et al. (2014) [42]	English	China mainland	ROS	NI	7	**✓**	**✗**	35	22	13	61.3	16/19	None	I-IV
Kim et al. (2013) [43]	English	Korea	ROS	1995–2008	8	**✓**	**✓**	165	74	91	NI	159/6	None	I-IV
Li et al. (2012) [44]	English	Taiwan	ROS	1999–2009	9	**✗**	**✓**	77	44	33	52.0	75/2	All received	I-III
Li et al. (2015) [45]	English	Taiwan	ROS	NI	8	**✓**	**✓**	105	59	46	54.0	103/2	None	I-IV
Lu et al. (2015) [46]	English	China mainland	ROS	2010–2012	8	**✓**	**✓**	148	94	54	59.0	114/34	None	I-III

*CP* clinicopathological, *CSS* cancer-specific survival, *DDE* demographic data extrapolated, *DFS* disease-free survival, *HR* hazard ratio, *IHC* immunohistochemistry, *M* multivariate, *NE* negative expression, *NI* no information, *NIT* neo-adjuvant induction therapy, *NOS* Newcastle-Ottawa Scale, *OR* odds ratio, *OS* overall survival, *PE* positive expression, *ROS* retrospective observational study, *U* univariate

**Table 2:** Baseline characteristics of included studies

Authors (Year)	Materials	Detection	Antibody	Dilution	Positive site	Cut-off value	Estimates	Extractions	Analysis	Endpoints	Follow-up
Boone et al. (2008) [38]	Paraffin-embedded tissue	IHC	Ser2448	1:50	Cytoplasm	20 % staining	OR	DDE	U	**––––**	**––––**
Chen et al. (2010) [39]	Paraffin-embedded tissue	IHC	Rabbit anti-mTOR	1:100	Cytoplasm	10 % staining	OR	DDE	U	**––––**	**––––**
Chuang et al. (2015) [40]	Paraffin-embedded tissue	IHC	Rabbit anti-mTOR	1:100	NI	Median H-score	OR, HR	DDE	U	OS	120 months
Hirashima et al. (2010) [41]	Paraffin-embedded tissue	IHC	Ser2448	1:50	Cytoplasm	10 % staining	OR, HR	Reported, DDE	U & M	OS, CSS	133 months
Hou et al. (2014) [42]	Paraffin-embedded tissue	IHC	Rabbit anti-mTOR	1:200	Cytoplasm	10 % staining	OR	DDE	U	**––––**	**––––**
Kim et al. (2013) [43]	Paraffin-embedded tissue	IHC	Ser2448	1:100	NI	5 % staining	OR, HR	Reported, DDE	U & M	OS, CSS	120 months
Li et al. (2012) [44]	Paraffin-embedded tissue	IHC	Ser2448	1:50	Cytoplasm	10 % staining	HR	Reported	M	OS, DFS	120 months
Li et al. (2015) [45]	Paraffin-embedded tissue	IHC	Ser2448	1:50	Cytoplasm	10 % staining	OR, HR	DDE	U	OS, DFS	146 months
Lu et al. (2015) [46]	Paraffin-embedded tissue	IHC	Rabbit anti-mTOR	1:100	Cytoplasm	25 % staining	OR, HR	Reported, DDE	U & M	OS, DFS	36 months

*CP* clinicopathological, *CSS* cancer-specific survival, *DDE* demographic data extrapolated, *DFS* disease-free survival, *HR* hazard ratio, *IHC* immunohistochemistry, *M* multivariate, *NE* negative expression, *NI* no information, *NIT* neo-adjuvant induction therapy, *NOS* Newcastle-Ottawa Scale, *OR* odds ratio, *OS* overall survival, *PE* positive expression, *ROS* retrospective observational study, *U* univariate

#### Study designs

There were 14 included studies reported from nine eligible articles, including eight studies focusing on the relationship between mTOR/p-mTOR expression and clinicopathological characteristics of ESCC and six studies analyzing the prognostic value of mTOR/p-mTOR expression in ESCC. All of these 14 included studies belong to retrospective observational studies [38–46], and they were published between 2008 and 2015 (Tables 1 and 2). Only one of them was finished in Chinese [39] and the others were published in English [38, 40–46].

#### Participants

This meta-analysis involved a total of 915 ESCC cases, including 502 Chinese patients from China mainland and Taiwan region (ratio = 54.9 %) [39, 40, 42, 44–46], 165 patients from Korea (ratio = 18.0 %) [43], 143 patients from Japan (ratio = 15.6 %) [41] and 105 patients from Netherland (ratio = 11.5 %) [38]. All of these patients were consecutively enrolled from 1989 to 2012. The sample size ranged from 34 to 165 across the included studies (Tables 1 and 2). Among these patients, there were 131 patients received NIT before esophagectomy and the remaining 784 patients underwent esophagectomy alone. In addition, the details for gender proportions, mean ages and clinical stages in each included study are also outlined in Tables 1 and 2.

#### Interventions

As for experimental materials, IHC was commonly used for staining mTOR/p-mTOR in paraffin-embedded specimens [38–46]. The cut-off definitions for positive expression of mTOR/p-mTOR and their corresponding positive sites varied across the current studies but overlapped for some common evaluations (Tables 1 and 2). There was also a substantial difference in antibody use (Ser2448 or Rabbit anti-mTOR antibody) and the corresponding dilution (ranged from 1:200 to 1:50) between studies (Tables 1 and 2). Given above reviews, mTOR/p-mTOR expression was detected in 462 patients, with the positive ratio of 50.5 %.

#### Outcome measures

To estimate the relationship between mTOR/p-mTOR expression and clinicopathological characteristics of ESCC, none of the eight relevant studies provided any statistical data derived from multivariate analysis but reported the demographic details [38–43, 45, 46]. The OR statistics incorporated into quantitative synthesis were commonly extrapolated from these demographics, which were based on univariate analysis (Tables 1 and 2).

To evaluate the prognostic significance of mTOR/p-mTOR expression in ESCC, seven multivariate statistics were directly reported from six included studies, including four HR statistics for OS [41, 43, 44, 46], two HR statistics for DFS [44, 46] and one HR statistic for CSS [43]. Besides, the additional four HR statistics could be extrapolated from the survival data based on univariate analysis, including two for OS [40, 45], one for CSS [41] and one for DFS [45]. In addition, the maximum follow-up period ranged from 36 to 146 months between studies (Tables 1 and 2).

### Risk of bias within studies

The quality level of all the included studies was graded by a NOS score, then listed by the number of stars (see the Additional file 2). Finally, these studies had a mean score of 7.8 (ranged from 7 to 9), indicating a fairly good quality level.

### Synthesis of results

#### Positive mTOR/p-mTOR expression and clinicopathological characteristics of ESCC

**Fig. 2:**
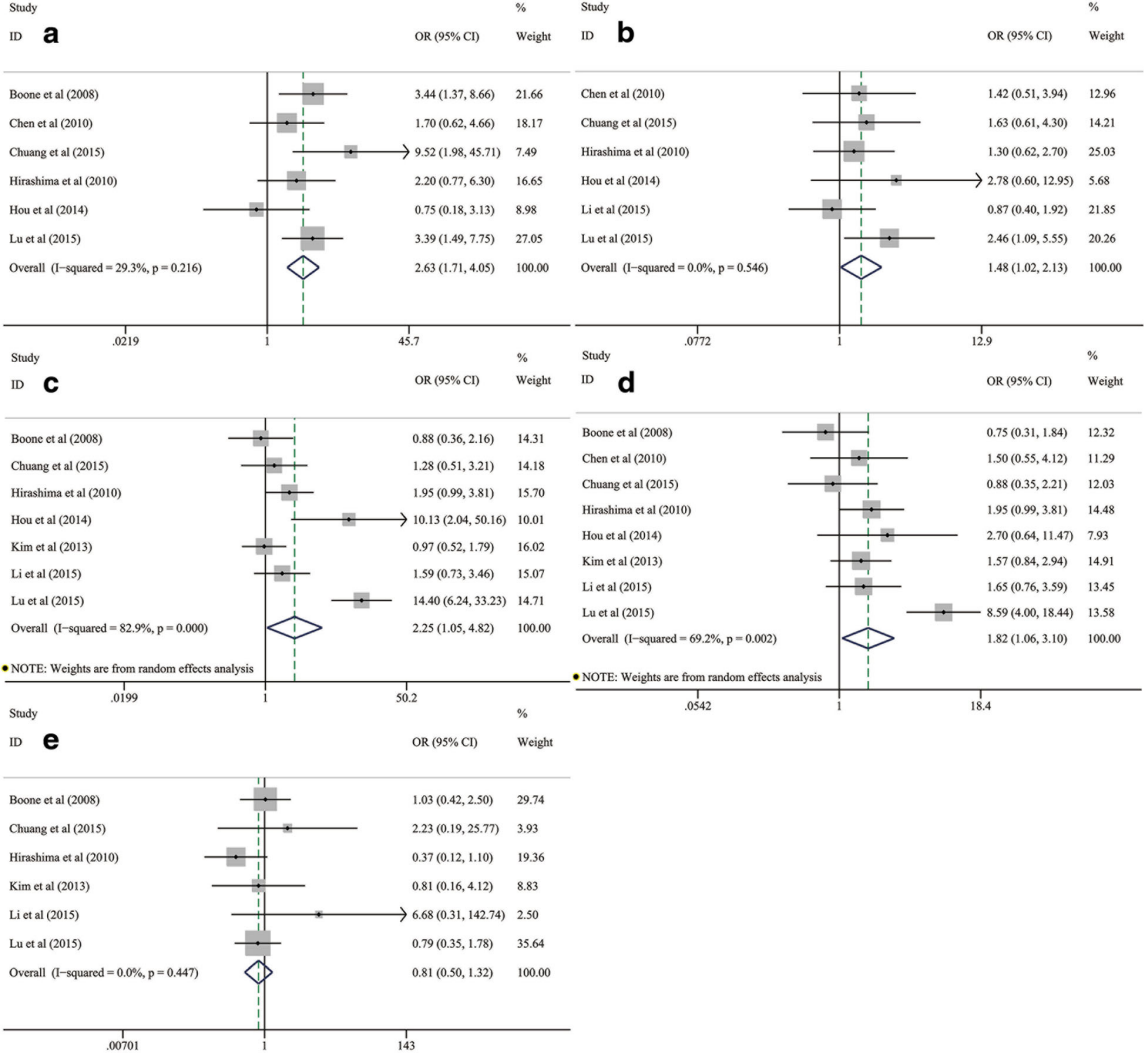
Meta-analysis on the relationship between mTOR/p-mTOR expression and clinicopathological characteristics of ESCC, including (**a**) differentiation degree, (**b**) tumor invasion, (**c**) TNM stage, (**d**) LNM and (**e**) genders. CI, confidence interval; ESCC, esophageal squamous cell carcinoma; LNM, lymph node metastasis; mTOR, mammalian target of rapamycin; p-mTOR, phosphorylated mammalian target of rapamycin; OR, odds ratio

In our meta-analysis, common clinicopathological variables of ESCC involved the gender, depth of tumor invasion (T factor), differentiation degree, TNM stage (III/IV vs I/II) and lymph node metastasis (LNM). Their pooled analyses showed that mTOR/p-mTOR expression was significantly associated with the worse outcomes for differentiation degree (OR: 2.63; 95 % CI: 1.71–4.05; *P* = 0.001; I^2^ = 29.3 %, *p* = 0.216; Table 3 and Fig. 2a), tumor invasion (OR: 1.48; 95 % CI: 1.02–2.13; *P* = 0.037; I^2^ = 0.0 %, *p* = 0.546; Table 3 and Fig. 2b), TNM stage (OR: 2.25; 95 % CI: 1.05–4.82; *P* = 0.037; I^2^ = 82.9 %, *p* < 0.001; Table 3 and Fig. 2c) and LNM (OR: 1.82; 95 % CI: 1.06–3.11; *P* = 0.029; I^2^ = 69.2 %, *p* = 0.002; Table 3 and Fig. 2d). However, mTOR/p-mTOR expression seemed to have no significant relationship to the genders of patients with ESCC (OR: 0.81; 95 % CI: 0.50–1.32; *P* = 0.396; I^2^ = 0.0 %, *p* = 0.447; Table 3 and Fig. 2e).

**Table 3:** Meta-analysis of relationships between mTOR/p-mTOR expression and clinicopathological characteristics of ESCC

Clinicopathological characteristics	*N*	No. samples			Heterogeneity (I^2^, *p*)	Model	OR with 95 % CI	*P* value	Publication bias		Conclusion
		Total	PE	NE					Begg (*p*)	Egger (*p*)	
Differentiation (G3 vs G1/G2)	6	568	285	283	I^2^ = 29.3 %, *p* = 0.216	Fixed	2.634 (1.714–4.047)	0.001	0.283	0.456	Significant
Depth of tumor invasion (T3/T4 vs T1/T2)	6	568	318	250	I^2^ = 0.0 %, *p* = 0.546	Fixed	1.477 (1.024–2.132)	0.037	0.452	0.355	Significant
TNM stage (III/IV vs I/II)	7	776	385	391	I^2^ = 82.9 %, *p <* 0.001	Random	2.248 (1.048–4.823)	0.037	0.087	0.216	Significant
Lymph node metastasis (Yes vs No)	8	838	418	420	I^2^ = 69.2 %, *p* = 0.002	Random	1.816 (1.062–3.105)	0.029	0.754	0.626	Significant
Gender (Male vs Female)	6	741	363	378	I^2^ = 0.0 %, *p* = 0.447	Fixed	0.811 (0.500–1.316)	0.396	0.371	0.273	Not significant

*CI* confidence interval, *ESCC* esophageal squamous cell carcinoma, *mTOR* Mammalian Target of Rapamycin, *N* reference count, *NE* negative expression, *OR* odds ratio, *PE* positive expression, *p-mTOR* Phosphorylated Mammalian Target of Rapamycin

#### Prognostic roles of mTOR/p-mTOR expression in patients with ESCC

**Fig. 3:**
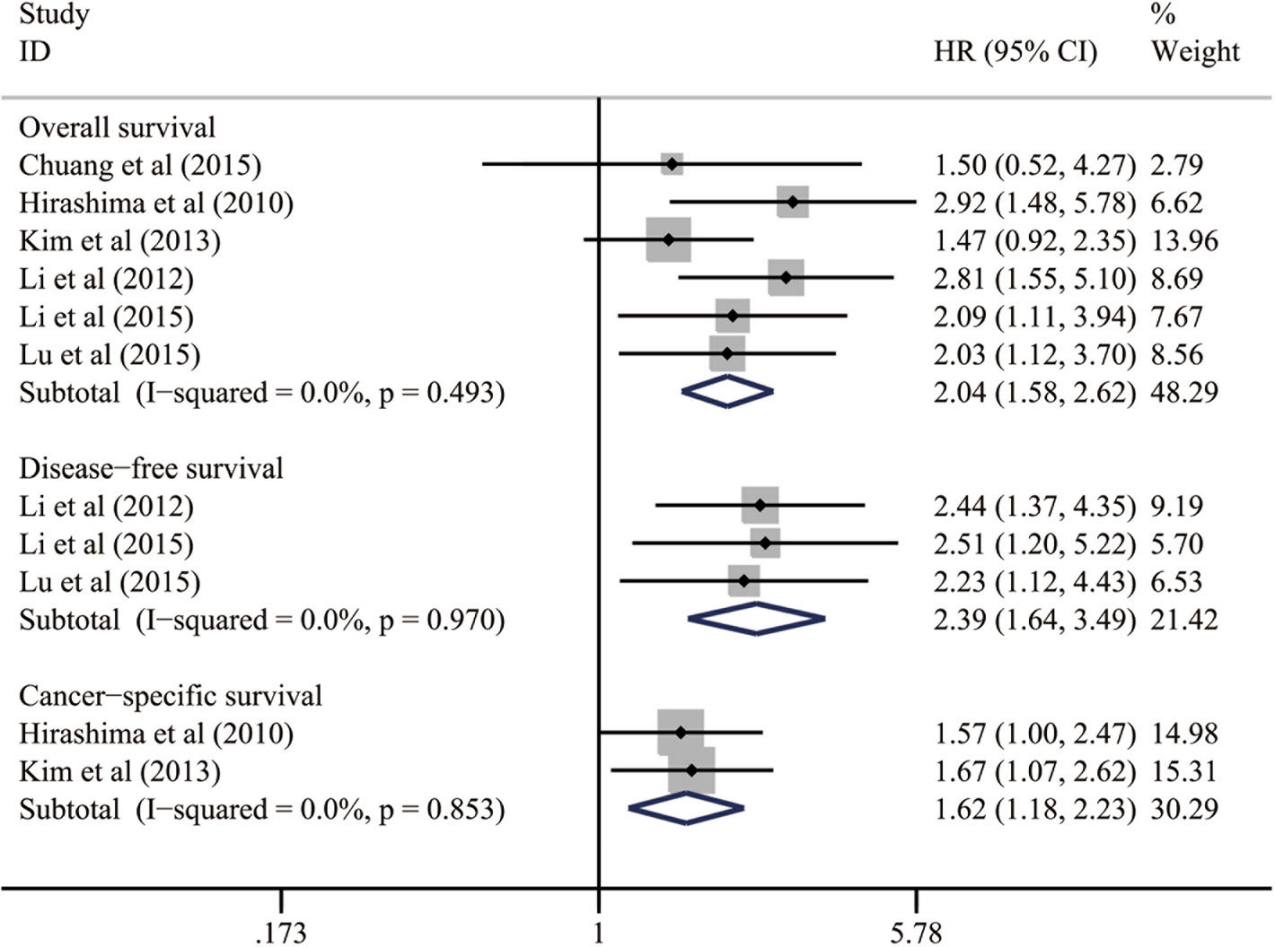
Meta-analysis on the prognostic significance of mTOR/p-mTOR expression for OS, DFS and CSS in patients with ESCC. CI, confidence interval; CSS, cancer-specific survival; DFS, disease-free survival; ESCC, esophageal squamous cell carcinoma; HR, hazard ratio; mTOR, mammalian target of rapamycin; p-mTOR, phosphorylated mammalian target of rapamycin; OS, overall survival

We performed a pooled analysis based on six included studies to evaluate the relationship between mTOR/p-mTOR expression and OS of ESCC patients [40, 41, 43–46]. The summarized estimates suggested that mTOR/p-mTOR expression was significantly correlated with the worse OS in patients with ESCC (HR: 2.04; 95 % CI: 1.58–2.62; *P* < 0.001; Table 4 and Fig. 3), without any heterogeneity (I^2^ = 0.0 %, *p* = 0.493).

**Table 4:** Meta-analysis of prognostic roles of mTOR/p-mTOR expression in patients with ESCC

Endpoint event	*N*	No. samples			Heterogeneity (I^2^, *p*)	Model	HR with 95 % CI	*P* value	Publication bias		Conclusion
		Total	PE	NE					Begg (*p*)	Egger (*p*)	
Overall survival	6	713	381	332	I^2^ = 0.0 %, *p* = 0.493	Fixed	2.036 (1.582–2.620)	<0.001	1.0	0.663	Significant
Disease-free survival	3	330	197	133	I^2^ = 0.0 %, *p* = 0.970	Fixed	2.390 (1.637–3.490)	<0.001	1.0	0.941	Significant
Cancer-specific survival	2	308	145	163	I^2^ = 0.0 %, *p* = 0.853	Fixed	1.620 (1.179–2.229)	0.003	1.0	NI	Significant

*CI* confidence interval, *ESCC* esophageal squamous cell carcinoma, *HR* hazard ratio, *mTOR* Mammalian Target of Rapamycin, *N* reference count, *NE* negative expression, *NI* no information, *PE* positive expression, *p-mTOR* Phosphorylated Mammalian Target of Rapamycin

Similarly, such significant relationships between mTOR/p-mTOR expression and unfavorable prognosis of ESCC were still statistically reliable within the pooled analyses of three studies for DFS outcomes (HR: 2.39; 95 % CI: 1.64–3.49; *P* < 0.001; I^2^ = 0.0 %, *p* = 0.970; Table 4 and Fig. 3) [44–46] and two studies for CSS outcomes (HR: 1.62; 95 % CI: 1.18–2.23; *P* = 0.003; I^2^ = 0.0 %, *p* = 0.853; Table 4 and Fig. 3) [41, 43]. All of the above integrated estimates indicated a strong predictive value of mTOR/p-mTOR expression for poor prognosis of ESCC.

#### Subgroup analyses on the prognostic value of mTOR/p-mTOR expression for OS in patients with ESCC

To further evaluate the prognostic significance of mTOR and p-mTOR in detail, all of six included studies addressing on the relationship between mTOR/p-mTOR expression and OS of ESCC cases were stratified into several subgroups according to the statistical analysis methods, cut-off values, follow-up periods and positive-staining sites [40, 41, 43–46].

**Fig. 4:**
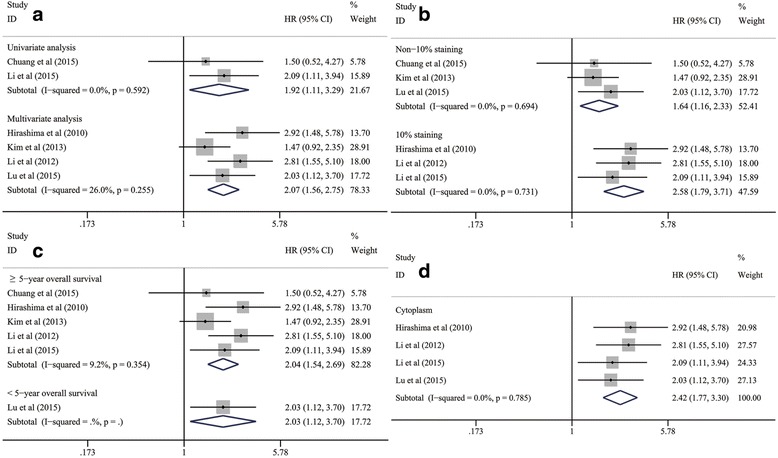
Subgroup analyses on the prognostic value of mTOR/p-mTOR expression for OS in subgroups of ESCC patients stratified by (**a**) statistical analysis, (**b**) cut-off values, (**c**) follow-up periods and (**d**) positive-staining sites. CI, confidence interval; ESCC, esophageal squamous cell carcinoma; HR, hazard ratio; mTOR, mammalian target of rapamycin; p-mTOR, phosphorylated mammalian target of rapamycin; OS, overall survival

A subgroup analysis was conducted in each of above subgroups. According to the pooled HR statistics, we found that the association between mTOR/p-mTOR expression and worse OS of ESCC still remained statistically prominent in all of the subgroups stratified by statistical analysis methods (multivariate data, HR: 2.07; 95 % CI: 1.56–2.75; *P* < 0.001; univariate data, HR: 1.92; 95 % CI: 1.11–3.29; *P* = 0.019; Table 5 and Fig. 4a), cut-off values (10 % staining, HR: 2.58; 95 % CI: 1.79–3.71; *P* < 0.001; non-10 % staining, HR: 1.64; 95 % CI: 1.16–2.33; *P* = 0.005; Table 5 and Fig. 4b), follow-up periods (≥5-year OS, HR: 2.04; 95 % CI: 1.54–2.69; *P* < 0.001; < 5-year OS, HR: 2.03; 95 % CI: 1.12–3.70; *P* = 0.020; Table 5 and Fig. 4c) and positive-staining sites (cytoplasmic staining, HR: 2.42; 95 % CI: 1.77–3.30; *P* < 0.001; Table 5 and Fig. 4d).

**Table 5:** Subgroup analyses for the relationship between mTOR/p-mTOR expression and OS of patients with ESCC

Subgroups	*N*	No. samples			Heterogeneity (I^2^, *p*)	Model	HR with 95 % CI	*P* value	Conclusion
		Total	PE	NE					
Subgroups stratified by statistical analysis									
Multivariate analysis	4	533	283	250	I^2^ = 26.0 %, *p* = 0.255	Fixed	2.071 (1.557–2.753)	<0.001	Significant
Univariate analysis	2	180	98	82	I^2^ = 0.0 %, *p* = 0.592	Fixed	1.915 (1.114–3.292)	0.019	Significant
Subgroups stratified by cut-off values									
10 % staining	3	325	174	151	I^2^ = 0.0 %, *p* = 0.731	Fixed	2.577 (1.788–3.714)	<0.001	Significant
Non-10 % staining	3	388	207	181	I^2^ = 0.0 %, *p* = 0.694	Fixed	1.644 (1.160–2.328)	0.005	Significant
Subgroups stratified by follow-up periods									
≥ 5-year OS	5	565	287	278	I^2^ = 9.2 %, *p* = 0.354	Fixed	2.037 (1.542–2.689)	<0.001	Significant
< 5-year OS	1	148	94	54	**––––**	**––––**	2.033 (1.117–3.701)	0.020	Significant
Subgroups stratified by positive-staining sites									
Cytoplasm	4	473	268	205	I^2^ = 0.0 %, *p* = 0.785	Fixed	2.416 (1.769–3.301)	<0.001	Significant
Nucleus or membrane	Given up because of the scarcity of available data								

*CI* confidence interval, *ESCC* esophageal squamous cell carcinoma, *HR* hazard ratio, *mTOR* Mammalian Target of Rapamycin, *N* reference count, *NE* negative expression, *OS* overall survival, *PE* positive expression, *p-mTOR* Phosphorylated Mammalian Target of Rapamycin

### Sensitivity analysis

**Fig. 5:**
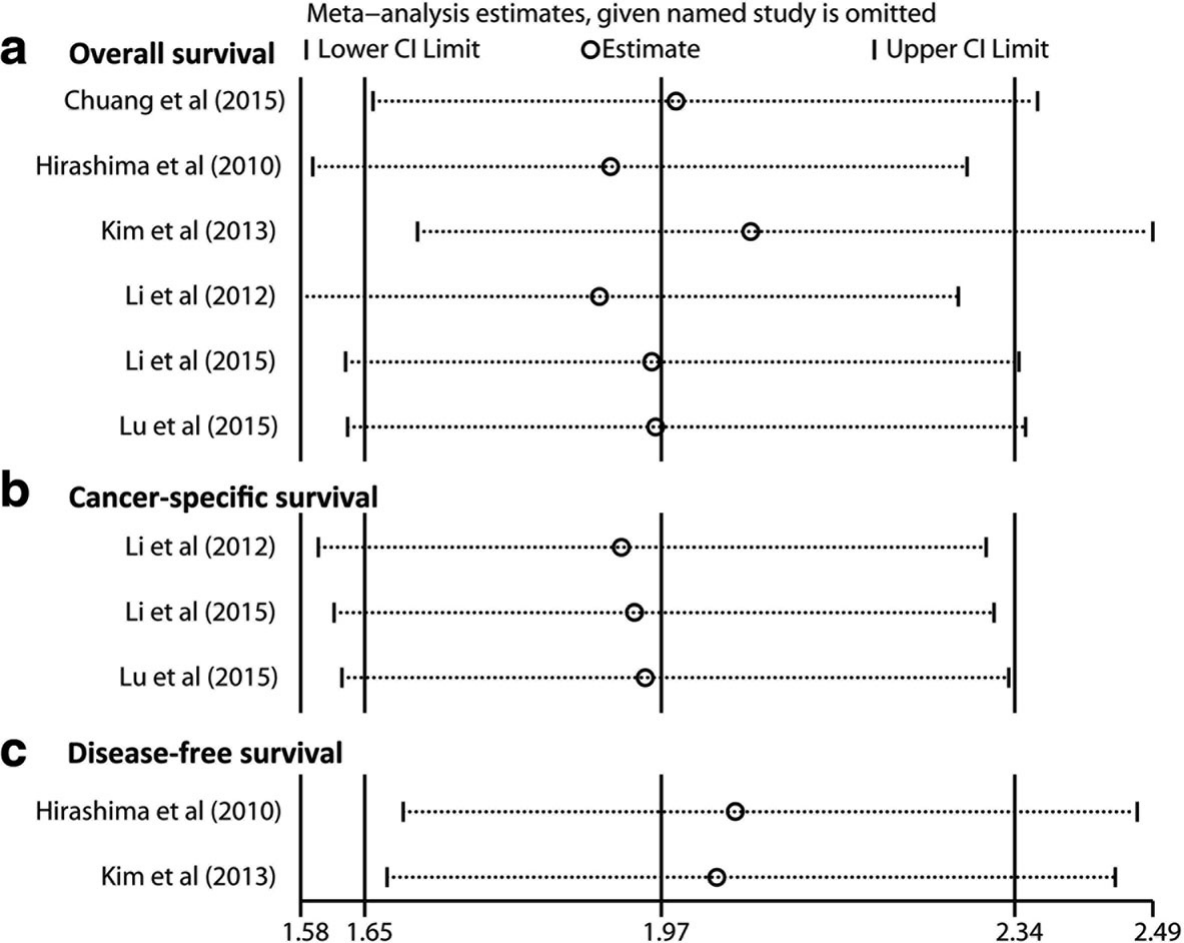
Sensitivity analysis on the prognostic value of mTOR/p-mTOR expression for (**a**) OS, (**b**) DFS and (**c**) CSS in patients with ESCC. CI, confidence interval; CSS, cancer-specific survival; DFS, disease-free survival; ESCC, esophageal squamous cell carcinoma; mTOR, mammalian target of rapamycin; p-mTOR, phosphorylated mammalian target of rapamycin; OS, overall survival

We conducted a sensitivity analysis to access the stability of three summarized HR outcomes revealing the prognostic value of mTOR/p-mTOR expression for OS, DFS and CSS in patients with ESCC. As Fig. 5a-c showed, no substantial variation was finally identified between the adjusted pooled HR and primary pooled HR by omitting the individual study sequentially. The strong robustness of prognostic significance of mTOR/p-mTOR expression in ESCC was thus confirmed.

### Publication bias

For assessments of publication bias, both the Begg’s *p* value and Egger’s *p* value are listed in Tables 2 and 3 (Begg’s funnel plots not shown). By estimating the corresponding *p* value, there was no evidence for significant publication bias detected by either Begg’s test or Egger’s test across all the included studies.

## Discussion

To the best of our knowledge, this is the first meta-analysis to demonstrate the prognostic significance of mTOR/p-mTOR expression and its relationship to the clinicopathological characteristics of ESCC. Our meta-analysis identified that positive expression of mTOR/p-mTOR was significantly correlated with the worse conditions on differentiation degree, depth of tumor invasion, LNM and TNM stage of ESCC but had no relationship to the genders. Remarkably, mTOR/p-mTOR expression was also significantly associated with the worse OS, DFS and CSS in patients with ESCC. Further subgroup analyses suggested that such significant relationships between mTOR/p-mTOR expression and poor OS of ESCC patients still remained statistically reliable in all of the subgroups stratified by statistical analysis methods, cut-off values, follow-up periods and positive-staining sites. All of these pooled analyses indicated that mTOR/p-mTOR could be a strong predictor for the poor prognosis of ESCC. Their stabilities were further confirmed by sensitivity analysis and no publication bias was detected across the current studies.

### Summary of evidence

In 1991, mTOR was firstly discovered as a mammalian homolog of the target of rapamycin (TOR) proteins in yeast mutants [47]. TOR is generally regarded as a target of the macrolide fungicide rapamycin according to the growth resistance of these mutants to rapamycin, and mTOR is the structurally and functionally conserved mammalian counterpart [47, 48]. Because the C-terminus of mTOR shares substantial homology to the catalytic domain of P13K, mTOR belongs to the P13K protein kinase family and is with a molecular weight of 289 kDa [49]. To date, the *mTOR* gene has frequently been examined in every eukaryote genome investigation.

It is commonly recognized that mTOR encompasses two distant functional protein complexes in the organism, mTORC1 and mTORC2 [14]. mTORC1 consists of mTOR, Raptor, mLST8, and two negative regulators, PRAS40 and DEPTOR. Meanwhile, mTORC2 contains mTOR, Rictor, mLST8, mSin1, Hsp70 and DEPTOR [11]. There is a huge difference in their sensitivities to the macrolide fungicide rapamycin. Recent studies suggest that the rapamycin-inhibition properties of mTOR mainly depends on the activation of mTORC1. The primary procedures of ribosomal biogenesis and protein synthesis are regulated by mTORC1 through the phosphorylation and activation of Ser2448 [50]. On the contrary, mTORC2 is deemed resistant to rapamycin. However, new evidence indicates that long-term treatment with rapamycin can disrupt the original mTORC2 assembly and sequester the newly synthesized mTOR molecules [51].

The mTOR signaling pathways strongly correlate with growth factors, nutrients and the energy availability underlying cell survival, growth, proliferation and death. Remarkably, mTOR acts as a “master switch” of further cellular catabolism and anabolism. In the classical upstream PI3K/Akt/mTOR pathway, stimulation of class I P13K activates its downstream effector AKT, and then leads to the phosphorylation of Ser2448 which plays a key role in activating the mTOR (p-mTOR) [52, 53]. Moreover, mTOR can be down-regulated through another upstream signaling pathway, the liver kinase B1/AMP-activated protein kinase/mTOR (LKB1/AMPK/mTOR) pathway. The anti-oncogene LKB1 directly phosphorylates the activation loop and increases the activity of AMPK kinase [54]. Activated AMPK has an exquisite sensitivity to very subtle changes in intracellular AMP levels and can directly inhibit the mTOR signals under energy stress [50, 55]. In the downstream mTOR signaling pathways, p-mTOR can improve the translation efficiency of 5’-TOR mRNA and accelerate protein synthesis by phosphorylating its downstream receptors during the translation process, such as elF4E and p70S6 kinase [56]. Thus, mTOR pathways can further regulate some physiological and pathological events through the activation and phosphorylation of various exogenous stimuli and essential signaling pathways [11].

On the basis of above molecular mechanisms, mTOR/p-mTOR expression has increasingly been identified to be involved in some cancers [57]. Aberrant activation of mTOR pathway induced by the loss of tumor suppressors and oncogene stimulation can significantly promote tumor growth, angiogenesis and metastasis [58]. The mutations in *mTOR* gene confer a probability of constitutive activation of mTOR signaling pathways, even under nutrient starvation conditions [59]. Other signaling components of the upstream and downstream mTOR pathways are also frequently altered during proliferation dysregulation, which is associated with poor cancer prognosis [50]. In addition, activated mTOR signals can also contribute to the development of several syndromes with benign tumors composed of architecturally disorganized but well-differentiated cells, such as Cowden’s syndrome, Peutz-Jeghers syndrome and tuberous sclerosis. These syndromes may further develop to the malignancy [60].

In recent years, numbers of clinical evidence have investigated the prognostic value of positive mTOR/p-mTOR expression in many common cancers, including ESCC. By integrating the outcome data from all the currently available studies, our meta-analysis showed that mTOR could be a strong biomarker for poor prognosis of ESCC, because that a prognostic marker would be considered of high predictive value for the negative prognosis if its HR value was larger than 2 [25, 28]. Remarkably, further subgroup analyses indicated that such relationships between mTOR/p-mTOR expression and worse survival of ESCC were not substantially altered by different endpoints, cut-off values and follow-up periods. Besides, we also had an attempt to estimate the relationship between mTOR/p-mTOR expression and several major clinicopathological of ESCC. Positive expression of mTOR/p-mTOR was found to be significantly associated with the worse conditions on tumor invasion, differentiation degree, TNM stage and LNM, all of which were critical factors resulting in the negative prognosis of ESCC. We speculated that the clinicopathological significance of mTOR/p-mTOR expression might be able to interpret its prognostic roles in ESCC to some extent.

Another issue worth to be discussed was the correlation between mTOR/p-mTOR expression and NIT sensitivity to ESCC. In this meta-analysis, only one study conducted by Li et al. [44] reported the survival data of 77 surgical patients followed by NIT. According to the multivariate analysis, p-mTOR was found to be independently associated with the response to NIT and prognosis of ESCC patients treated with NIT. Their laboratorial evidence also indicated that inhibition of mTOR could sensitize ESCC cell lines to chemotherapy, suggesting that the mTOR inhibitor could enhance the efficacy of NIT. However, as the researchers suggested, the validity of these findings might be limited by relatively short follow-up periods and small sample availability. Anyway, the significance of mTOR activation in NIT sensitivity and its impact on the prognosis of ESCC should be further evaluated by more large-scale studies with prolonged follow-up periods in the future.

### Limitations

We noticed that the following five fields of bias risks might cause adverse effects on the validity of pooled estimates within our meta-analysis. These major limitations should be acknowledged and seriously considered in the clinical practices.

First of all, our pooled analyses on the prognostic significance of mTOR/p-mTOR expression and its relationship to clinicopathological characteristics of ESCC were based on only 915 ESCC cases enrolled from 14 retrospective observational studies [38–46]. It is generally proposed that multivariate analysis using Cox proportional hazards model or logistic regression is usually adopted to eliminate the potential bias risks from other confounding factors in observational studies [25–27, 29]. In this meta-analysis, no multivariate OR statistic was reported to assess the relationship between mTOR/p-mTOR expression and clinicopathological features of ESCC. Furthermore, there were two HR statistics extrapolated from the published survival details based on univariate analysis and incorporated into the prognostic assessments. Thus, we doubted that the accuracy of overall pooled estimates for OS, DFS and CSS might be slightly attenuated by some insufficiently eliminated confounders that could affect the prognosis of ESCC, such as TNM stages, tumor invasion, differentiation degree and LNM. These possible parameters might interfere the identification of actual roles of mTOR and p-mTOR in ESCC, although a strong linkage between mTOR/p-mTOR expression and their unfavorable conditions had been revealed by our meta-analysis. Therefore, the validity and accuracy of all the summarized outcomes should be further verified and modified in the future multivariate analyses without any bias risk from other confounding factors.

Second, there is a substantial variation in the proportion of positive samples across the included studies, which ranged from 24.8 % to as high as 62.9 % [38, 42]. We suspected that the huge heterogeneity existed within the cut-off values for positive expression of mTOR/p-mTOR might be the most compelling explanations for this finding. Because cut-off definitions for positive mTOR expression varied notably from 5 % staining to 25 % staining of cancer cells between studies, an unavoidable deviation originated from heterogeneous criteria of evaluation could negatively affect the validity of pooled estimates. Another one major reason worthy of our attentions was the different antibodies and dilutions used in the experimental IHC techniques (Tables 1 and 2). A scarcity of unified IHC methods could also cause adverse effects on the homogeneity level of included studies [25]. Given such concerns, all of above aspects of limitations must be judiciously evaluated when interpreting our summarized outcomes correctly.

Third, we noticed that the study years of included studies ranged from 1989 to 2012, and the ESCC staging criteria had some changes during this time-frame of nearly 25 years. The pathological stages of ESCC were determined according to the latest seventh edition of American Joint Committee on Cancer (AJCC) staging system in five eligible articles [40, 43–46]. However, the earliest study conducted by Boone et al. [38] was published in 2008 and staged the ESCC according to the previous sixth edition AJCC criteria. Compared to the sixth edition of AJCC staging system, the updated seventh criteria has a great improvement in the refinement of LNM classification and also enrich the traditional TNM staging by adding evaluations for histological subtypes, differentiation degree and tumor locations [61]. Therefore, the variations of ESCC staging could be one important source of bias when synthesizing the current evidence in our meta-analysis.

Fourth, it has been generally recognized that studies reporting beneficial intervention effects or a larger effect size are more likely to be published, while an equal amount of data towards the other directions may remain unpublished [29]. This phenomenon suggests that we cannot avoid the potential publication bias between studies included into a meta-analysis. However, less than 10 included studies can lead to a large decline on the efficacy of publication bias tests, resulting in the potentially misleading evidence for publication bias [25–29]. As for our meta-analysis, no more than 10 studies were included into each analysis for either clinicopathological characteristics or prognosis of ESCC. We suspected that potential publication bias might still exist across the included studies, although no significant evidence could be validly detected up to now.

Finally, we gave up to stratify the enrolled patients according to their nations and perform a subgroup analysis to further evaluate the potential ethnic differences in prognostic roles of mTOR/p-mTOR expression for ESCC, because all of the included studies were conducted among East-Asian populations [40, 41, 43–46]. No study assessing the prognostic value of mTOR/p-mTOR in Western ESCC patients was identified but only one study reported by Boone et al. [38] analyzed the relationship between mTOR/p-mTOR expression and clinicopathological features of 105 ESCC patients from the Netherlands. In general, ESCC occurs much more frequently in patients considered with a low socioeconomic status and living conditions because they receive little medical treatment and health care [62]. That may be a reason for the scarcity of available evidence from developed Western countries. Recently, a large genome-wide association study on ESCC has identified several susceptibility genes in Chinese populations [63]. However, no genetic susceptibility to ESCC has been identified among Caucasians until now. The potential effects of susceptibility genes on the activity of mTOR remain unclear. Therefore, our findings should be judiciously considered in the clinical settings of Western nations. More well-designed clinical reports were urgently required to clarify the prognostic value of mTOR in Caucasian patients with ESCC.

## Conclusions

In conclusion, our meta-analysis demonstrated that the positive expression of mTOR and p-mTOR was significantly correlated with the unfavorable outcomes on the depth of tumor invasion, TNM stage, differentiation degree and LNM. Furthermore, mTOR could serve as an independent predictor for the poor prognosis of ESCC. Some controversies and limitations are still not well-resolved in this meta-analysis. More high-quality worldwide studies performing a multivariate analysis based on large sample size will be very helpful for further verifying and modifying our current findings in the future.

## Additional files

Additional file 1: Summary of electronic literature retrieval. (DOCX 14 kb)
Additional file 2: Quality assessments of included studies. (DOCX 14 kb)

## Abbreviations

AJCC: American joint committee on cancer
Akt: v-akt murine thymoma viral oncogene homolog 1
AMPK: AMP-activated protein kinase
CI: Confidence interval
CSS: Cancer-specific survival
DDE: Demographic data extrapolated
DFS: Disease-free survival
elF4E: Eukaryotic translation factor 4E
ESCC: Esophageal squamous cell carcinoma
HR: Hazard ratio
IHC: Immunohistochemistry
LKB1: Liver kinase B1
LNM: Lymph node metastasis
mTOR: Mammalian target of rapamycin
NE: Negative expression
NI: No information
NIT: Neo-adjuvant induction therapy
NOS: Newcastle-Ottawa scale
OR: Odds ratio
OS: Overall survival
p70S6 kinase: p70 ribosomal S6 kinase
PE: Positive expression
PI3K: Phosphatidylinositol 3-kinase
p-mTOR: Phosphorylated mammalian target of rapamycin
PRISMA: Preferred reporting items for systematic reviews and meta-analyses
ROS: Retrospective observational study
RR: Relative risk
TOR: Target of rapamycin.
