# Supplementary material for: Clinicopathological and prognostic significance of mTOR and phosphorylated mTOR expression in patients with esophageal squamous cell carcinoma: a systematic review and meta-analysis
Source: BMC Cancer. 2016 Nov 11;16:877. doi: 10.1186/s12885-016-2940-7 (PMC5106813; doi:10.1186/s12885-016-2940-7)
Supplement: Additional file 1: — Summary of electronic literature retrieval. (DOCX 14 kb) [file 12885_2016_2940_MOESM1_ESM.docx]

***Additional file 1***

***Summary of electronic literature retrieval***

***PubMed search strategy***

| **Searches** | **Search details** | **Items found** |
| --- | --- | --- |
| **#1** | **Search** (((("oesophageal cancer"[All Fields] OR "esophageal neoplasms"[MeSH Terms] OR ("esophageal"[All Fields] AND "neoplasms"[All Fields]) OR "esophageal neoplasms"[All Fields] OR ("esophageal"[All Fields] AND "cancer"[All Fields]) OR "esophageal cancer"[All Fields]) OR (esophageal[All Fields] AND ("carcinoma"[MeSH Terms] OR "carcinoma"[All Fields]))) OR ("oesophageal neoplasm"[All Fields] OR "esophageal neoplasms"[MeSH Terms] OR ("esophageal"[All Fields] AND "neoplasms"[All Fields]) OR "esophageal neoplasms"[All Fields] OR ("esophageal"[All Fields] AND "neoplasm"[All Fields]) OR "esophageal neoplasm"[All Fields])) OR ("esophageal neoplasms"[MeSH Terms] OR ("esophageal"[All Fields] AND "neoplasms"[All Fields]) OR "esophageal neoplasms"[All Fields] OR ("esophageal"[All Fields] AND "malignancy"[All Fields]) OR "esophageal malignancy"[All Fields])) AND ("tor serine-threonine kinases"[MeSH Terms] OR ("tor"[All Fields] AND "serine-threonine"[All Fields] AND "kinases"[All Fields]) OR "tor serine-threonine kinases"[All Fields] OR ("mammalian"[All Fields] AND "target"[All Fields] AND "rapamycin"[All Fields]) OR "mammalian target of rapamycin"[All Fields]) | **72** |
| **#2** | **Search** (((("oesophageal cancer"[All Fields] OR "esophageal neoplasms"[MeSH Terms] OR ("esophageal"[All Fields] AND "neoplasms"[All Fields]) OR "esophageal neoplasms"[All Fields] OR ("esophageal"[All Fields] AND "cancer"[All Fields]) OR "esophageal cancer"[All Fields]) OR (esophageal[All Fields] AND ("carcinoma"[MeSH Terms] OR "carcinoma"[All Fields]))) OR ("oesophageal neoplasm"[All Fields] OR "esophageal neoplasms"[MeSH Terms] OR ("esophageal"[All Fields] AND "neoplasms"[All Fields]) OR "esophageal neoplasms"[All Fields] OR ("esophageal"[All Fields] AND "neoplasm"[All Fields]) OR "esophageal neoplasm"[All Fields])) OR ("esophageal neoplasms"[MeSH Terms] OR ("esophageal"[All Fields] AND "neoplasms"[All Fields]) OR "esophageal neoplasms"[All Fields] OR ("esophageal"[All Fields] AND "malignancy"[All Fields]) OR "esophageal malignancy"[All Fields])) AND mTOR[All Fields] | **83** |

***EMBASE (via Ovid interface) search strategy***

| **Searches** | **Search details** | **Items found** |
| --- | --- | --- |
| **#1** | **Search** ((esophageal cancer or esophageal carcinoma or esophageal neoplasm or esophageal malignancy) and mammalian target of rapamycin).af. | **56** |
| **#2** | **Search** ((esophageal cancer or esophageal carcinoma or esophageal neoplasm or esophageal malignancy) and mTOR).af. | **63** |

***The Web of Science (via campus network of Sichuan University) search strategy***

| **Searches** | **Search details** | **Items found** |
| --- | --- | --- |
| **#1** | **Search** TS=((esophageal cancer OR esophageal carcinoma OR esophageal neoplasm OR esophageal malignancy) AND mammalian target of rapamycin) | **96** |
| **#2** | **Search** TS=((esophageal cancer OR esophageal carcinoma OR esophageal neoplasm OR esophageal malignancy) AND mTOR) | **151** |
