# Supplementary material for: Clinicopathological and prognostic significance of mTOR and phosphorylated mTOR expression in patients with esophageal squamous cell carcinoma: a systematic review and meta-analysis
Source: BMC Cancer. 2016 Nov 11;16:877. doi: 10.1186/s12885-016-2940-7 (PMC5106813; doi:10.1186/s12885-016-2940-7)
Supplement: Additional file 2: — Quality assessments of included studies. (DOCX 14 kb) [file 12885_2016_2940_MOESM2_ESM.docx]

***Additional file 2***

***NOS scoring of included studies***

| **Authors (year)** | **Selection** | **Comparability** | **Exposure** | | | **Total score** |
| --- | --- | --- | --- | --- | --- | --- |
|  |  |  | **Assessment of outcome** | **follow-up long enough for outcomes** | **Adequacy of follow-up of cohorts** |  |
| Boone et al (2008) [38] | **4** | **1** | **1** | **0** | **1** | **7** |
| Chen et al (2010) [39] | **4** | **1** | **1** | **0** | **1** | **7** |
| Chuang et al (2015) [40] | **4** | **1** | **1** | **1** | **1** | **8** |
| Hirashima et al (2010) [41] | **4** | **2** | **1** | **1** | **1** | **9** |
| Hou et al (2014) [42] | **4** | **1** | **1** | **0** | **1** | **7** |
| Kim et al (2013) [43] | **4** | **2** | **0** | **1** | **1** | **8** |
| Li et al (2012) [44] | **4** | **2** | **1** | **1** | **1** | **9** |
| Li et al (2015) [45] | **4** | **1** | **1** | **1** | **1** | **8** |
| Lu et al (2015) [46] | **4** | **1** | **1** | **0** | **1** | **8** |

NOS, Newcastle-Ottawa Scale
